# *Mycobacterium abscessus complex*: A Review of Recent Developments in an Emerging Pathogen

**DOI:** 10.3389/fcimb.2021.659997

**Published:** 2021-04-26

**Authors:** Laura Victoria, Amolika Gupta, Jose Luis Gómez, Jaime Robledo

**Affiliations:** ^1^ Laboratory of Bacteriology and Mycobacteria, Corporación para Investigaciones Biológicas (CIB), Medellín, Colombia; ^2^ Escuela de Ciencias de la Salud, Universidad Pontificia Bolivariana, Medellín, Colombia; ^3^ Pulmonary, Critical Care and Sleep Medicine Section, Yale University School of Medicine, New Haven, CT, United States

**Keywords:** *Mycobacterium abscessus*, lung, infection, resistance, treatment, epidemiology

## Abstract

*Mycobacterium abscessus complex* (MABC*)* is one of the most clinically relevant species among nontuberculous mycobacteria. MABC’s prevalence has increased over the last two decades. Although these changes can be explained by improvements in microbiological and molecular techniques for identifying species and subspecies, a higher prevalence of chronic lung diseases may contribute to higher rates of MABC. High rates of antimicrobial resistance are seen in MABC, and patients experience multiple relapses with low cure rates. This review aims to integrate existing knowledge about MABC epidemiology, microbiological identification and familiarize readers with molecular mechanisms of resistance and therapeutic options for pulmonary infections with MABC.

## Introduction

The prevalence of pulmonary infections caused by non-tuberculous mycobacteria (NTM) has increased over the past three decades (Kendall and Winthrop, 2013; Stout et al., 2016). In 1987, the US centers for disease control and prevention (CDC) estimated the NTM disease rate of 1.8/100.000 (Kendall and Winthrop, 2013). Data from North American studies between 2006 and 2012 suggested a disease rate of 5 to 10 per 100,000 (Prevots and Marras, 2015). Of the NTM causing pulmonary infections, *Mycobacterium abscessus complex* (*MABC*) is one of the most significant mycobacterial isolates associated with pulmonary infections (Schiff et al., 2019), particularly in patients with cystic fibrosis (Abdalla et al., 2015). Among the rapidly growing mycobacteria (*RGM*), *MABC* is considered the most pathogenic of this group of pathogens (Medjahed et al., 2019). In particular, *MABC* is associated with intrinsic and acquired resistance to most anti-mycobacterial agents, including macrolides (Lopeman et al., 2019).

### Taxonomy

Since *MABC* was first isolated in 1952 and was considered a new species of NTM (Lopeman et al., 2019), nomenclature has changed multiple times. In 1972, *MABC* was designated as *M. chelonae subspecies abscessus* but in 1992 thanks to DNA hybridization *MABC* was recognized as an independent species (Lee et al., 2015; Lopeman et al., 2019). In 2006, two new species *M. massiliense* and *M. bolletii* were described as novel and closely related to *M. abscessus* based on *the rpoB* gene sequence (Tortoli et al., 2016). However, since the three of them have more than 70% relatedness (based on DNA-DNA hybridization), *M. massiliense, M. bolletii*, and *M. abscessus* were presented as subspecies and the combinations of the three subspecies were known as *Mycobacterium abscessus complex (MABC)* (Tortoli et al., 2016).

Taxonomy classification has been controversial due to the significant similarities between *M.* *massiliense and M. bolletii*. In 2011, some authors considered that *M. massiliense and M. bolettii* should be classified as a single subspecies: *M. abscessus subsp. bolletii.* In 2013, whole-genome sequencing-based phylogenetic studies supported their differentiation in three distinct subspecies belonging to *MABC (* Lopeman et al., 2019). Additionally, the three subspecies are phenotypically divergent with clinically relevant impact which supports the importance of their differentiation as described by Tortoli et al., in 2016 (Tortoli et al., 2016; Lopeman et al., 2019). Currently, the three subspecies fulfill the criteria to be considered subspecies (Tortoli et al., 2018).

Recently, it was proposed to divide the genus *Mycobacterium* into 5 distinct genera: *Mycobacterium*, *Mycolicibacterium*, *Mycolicibacter*, *Mycolicibacillus* and *Mycobacteroides.* The basis for this division is the presence of specific molecular markers shared within a group of related taxa (Turenne, 2019). *Mycobacteroides*, proposed as new genus distinguished by 51 unique molecular markers, is also known as *rapidly growing mycobacteroides* and includes *M. abscessus-M. chelonae clade* (Gupta et al., 2018). The changed name of the genus proposed that affects the species name as *Mycobacteroides abscessus*, it is now considered a homotypic synonym of the species. Nevertheless, this approach is not widely yet and mycobacterium may continue to be used for *MAB* taxonomy.

### Trends in Pulmonary Disease

*MABC* is the most common etiological agent of pulmonary disease caused by RGM, representing 3-13% of all NTM-PD (Honda et al., 2018). *M. abscessus* is the most common isolate, followed by *M. massiliense* and *M. bolletii (* Koh et al., 2014). However, the proportion of the three subspecies may vary according to geographical distribution (Koh et al., 2011).

In high burden areas of TB, NTM-PD has often been mistaken as multidrug-resistant tuberculosis (MDR-TB) (Shahraki et al., 2015). Patients treated for MDR-TB may have NTM disease (12-30% of cases) (Nishiuchi et al., 2017). In a referral center in Brazil, 79% of patients had been treated empirically for TB for up to 6 months before the diagnosis of NTM disease was confirmed, evidencing a frequent misdiagnosis of NTM-PD (Prevots and Marras, 2015).

A systematic review that included 38,686 NTM isolates showed that *MABC* was frequently isolated in Asia (16%) and Oceania (12%) and less frequently in South America (5.7%) North America (3.2%) and Europe (2.9%). Epidemiology updates of NTM reveal that the isolation frequency and clinical relevance of NTM in different world regions were *MAC* (88%) *M*. *Kansassii* (78%) and *MABC* (61%) (Zweijpfenning et al., 2018).

### Environmental Sources

NTM have been isolated from water tanks, hot tubes, swimming pools, medical devices such as endoscopes, seawater, house dust, and livestock, making exposure extremely common (Somoskovi, 2014; Halstrom et al., 2015; Loret and Dumoutier, 2019).

Environmental updates reveal that *MABC* is resistant to high chlorine levels that allows this mycobacterium to survive in drinking water (Jones et al., 2019). It is also resistant to disinfectants such as organic-mercurial substances and alkaline glutaraldehyde (Brown-Elliott and Wallace, 2002). *MABC* can form biofilms and resist high temperatures, which allow it to survive in water systems (Howard, 2013; Honda et al., 2018; Jones et al., 2019).

### Risk Factors For *MABC-PD*

In patients with cystic fibrosis (CF), the estimated prevalence of *MABC-PD* is 13% in Europe and 16% in US (Harris and Kenna, 2014). Gastroesophageal disease, achalasia, and recurrent vomiting are conditions associated with recurrent aspiration of gastric contents, all reversible risk factors for *MABC-PD (* Jarand et al., 2011). Prior mycobacterial infection is a well-known risk factor for *MABC-PD* with previous or coexisting *MAC* as high as 55.1% (Jarand et al., 2011; Tung et al., 2015). Although there are no specific immune defects associated with *MABC-PD*, autosomal recessive INF-γ R1 deficiency has been described as a risk factor (Bustamante et al., 2014). A summary of related conditions is described in **Table 1**.

**Table 1:** Risk factors associated with non-tuberculous mycobacterial pulmonary disease.

NTM-PD^a^	MABC-PD^b^
Chronic obstructive pulmonary disease	Pre-existing pulmonary disease
Alpha-1-antitrypsin deficiency	Cystic fibrosis
Pneumoconiosis	Bronchiectasis
Ciliary dyskinesia	Gastroesophageal reflux
Prior granulomatous disease	Achalasia
Thoracic skeletal abnormalities	Lipoid pneumonia
Macrophage dysfunction	Solid-organ cancer
Biologic agents	History of Mycobacterial disease
Steroid use	(i.e. TB and NTM)
CD4 lymphopenias	
Hematologic malignancy	
Mendelian susceptibility to NTM^c^	

a Multiple host susceptibility factors have been described for all NTM species in pulmonary disease, including MABC (Prevots and Marras, 2015; Stout et al., 2016; Drummond and Kasperbauer, 2019; van Ingen et al., 2019).

b Some risk factors appear to be highly associated with MABC-PD (Jarand et al., 2011; Howard, 2013; van Ingen et al., 2019; Bryant et al., 9877).

c Inherited conditions characterized by a predisposition to clinical disease by environmental mycobacteria in otherwise healthy individuals. IFN-γR1 deficiency is associated with early-onset disseminated infections by species such as M. chelonae, M. fortuitum, M. mageritense, M. peregrinum, M. smegmatis, and M. scrofulaceum. Patients with IFN-γR2 deficiency present multibacillary granulomas by M. abscessus, M. avium, M. fortuitum and M. simiae (Bustamante et al., 2014).

### Transmission

There is no substantial evidence for person-to-person transmission or direct transmission from animals, but cases of infection occurring at the same location suggest a common source (Howard, 2013; Somoskovi, 2014; Bryant et al., 9877). Whole-genome sequencing (WGS) studies of outbreaks occurring in CF patients demonstrated clonality among isolates, suggesting that person-to-person transmission is a possible way of transmission (Howard, 2013). Additional evidence showed a few dominant clones spreading globally in the CF community (Ravnholt et al., 2018; Bryant et al., 9877). Transmission updates suggest that MABC is transmissible between CF patients in the hospital setting, including the transmission of macrolide-resistant isolates (Köser et al., 2014; Bryant et al., 9877). The finding that resistant strains might be transmissible has created a shift in our knowledge of MABC transmission (Ravnholt et al., 2018; Yoshida et al., 2018), and raised concerns about the clinical impact associated with global propagation of these strains.

### Immune Response, Virulence and Genomic Features

Mycobacterial virulence depends on the ability of bacteria to invade, grow, and persist in the host. Type I interferons (IFN-I) such us IFNβ and IFNα play a critical role during *MABC* infection (Ruangkiattikul et al., 2019). Activation of toll-like receptors (TLR), specifically TLR2 and TLR4 results in MyD88/TRIF/IRF3 dependent IFN-I induction (Ruangkiattikul et al., 2019; Peignier and Parker, 2021). IFN-I production in infected macrophages activates inducible nitric oxide synthase (NOS2) and nitric oxide (NO) production which can kill or induce dormancy. IFN-I plays a key role to induce NO production and intensify the ability of macrophages to clear MABC infection (Ruangkiattikul et al., 2019). Data suggest that persistence of *MABC* infections in CF patients could be explained by a limited IFN-I response. Interferon gamma (IFN-γ), a type II IFN is also required to control *MABC* infection, there have been several reports of disseminated disease in patients with defects in the IFN-γ pathway (Rottman et al., 2007).

*MABC* induces a strong TLR2 mediated TNFα response, a key inflammatory cytokine that mediates mycobacterial killing, host defense and granuloma formation (Bernut et al., 2016; Kim et al., 2017). TNF/IL8 signaling pathway activates macrophage bactericidal activity, restrict extracellular growth, and increase neutrophil recruitment and mobilization which is required for granuloma formation (Bernut et al., 2016). It has also been shown that highly virulent isolates stimulate TNF secretion by macrophages (Medjahed et al., 2019). Impairment of this pathway correlates with disseminated disease and lethal infection (Bernut et al., 2016; Bernut et al., 2019). However, excessive levels of TNF-a can lead to detrimental effects in the host secondary to tissue damage (Kim et al., 2017).

*MABC* from clinical isolates have shown a rough vs smooth colony morphology and is able to shift between these forms (Medjahed et al., 2019). A zebrafish experimental model has been recently used for the study of *MABC* virulence, using this model, researchers observed that the rough morphology forms serpentine cords and large bacterial clumps, the formation of large cords allows this mycobacterium to escape the immune system as extremely large size of cords might prevent *MABC* from being internalized as they are larger in size than macrophages (Bernut et al., 2014; Bernut et al., 2017). This promotes spreads to other tissues and extracellular replication that results in abscess formation and tissue damage (Bernut et al., 2014). The rough morphology is also associated with increased apoptosis leading to increases in extracellular bacteria and promotion of cord formation (Bernut et al., 2014). Interestingly, CFTR (CF transmembrane conductance regulator) defects, such as those seen in CF, has been associated with impaired NADPH oxidase production which leads to increase intracellular growth and reduce neutrophil chemotaxis, compromising granuloma integrity (Bernut et al., 2019).

The absence of glycopeptidolipid (GLP), a molecule in the outer surface of the cell wall, has been associated with rough colony morphology. Defects in the mmpL4b gene are associated with the loss of GLP, leading to conversion to a rough phenotype showing morphological plasticity (Nessar et al., 2011; Bernut et al., 2016). The GLP loss unmask lipoproteins that produce a strong inflammatory response (Nessar et al., 2011). This correlates with the fact that hypervirulence has been observed in the rough morphology vs smooth morphology (Bernut et al., 2014). Rough morphology induces higher levels of TNF-I (Ruangkiattikul et al., 2019). Smooth morphology induces lower levels of IFN-I which favors persistence (Ruangkiattikul et al., 2019). Intramacrophage survival of the smooth morphology is also explained by phago-lysosomal fusion block and resistance to apoptosis, the phagosome shows membrane disruption at early stages of infection leading to phagosome- cytosol communication and phagosomal escape allowing extracellular replication (Bernut et al., 2017). Phagosomal escape is independent of ESX-1 mechanism as *MABC* only has two ESX gene clusters (ESX-3 and ESX-4) which differs from TBC (Kim et al., 2017). Cord formation is a unique and new immune evasion mechanism in *MABC* infection.

The three subspecies *of MABC* are separated based on multilocus sequencing of housekeeping genes (Harris and Kenna, 2014). Unlike other *RGM, MABC* has a single ribosomal RNA operon, making the phenotypic expression of single mutation more likely. The reference strain (ATCC19977) contains a full genome sequence of 5.1 Megabases (Mb) with a high number of conserved genes (Cortes et al., 2010; Medjahed et al., 2019). It includes an 81- Kb full-length prophage, five insertion elements, and a 23 Kb- mercury resistance plasmid, which has been associated with infection in young patients with CF (Cortes et al., 2010). The resistant plasmid is highly similar to an episome present in *M. Marinum*, suggesting that these species may have exchanged the plasmid (Medjahed et al., 2019). *MABC* contains unique genes not present in other mycobacteria that appear to have been acquired by horizontal gene transfer from different species such as *pseudomonas* sp. and *streptomyces* sp (Howard, 2013; Medjahed et al., 2019).. These shared genes are thought to contribute to the pathogenesis of *Pseudomonas* sp. and *MABC-PD* facilitating respiratory tract colonization in CF patients (Nessar et al., 2011).

### Clinical Disease

Updates reveal that there are two presentations of NTM pulmonary infections. One is an upper lobe fibrocavitary form while the other is a nodular bronchiectatic form (NB). The upper lobe fibrocavitary form is characterized by cavitary lesions similar to tuberculosis and usually occurs in older males with underlying lung disease (Shin et al., 2013; Moon et al., 2019). It is rapidly progressive and can lead to lung destruction in a short period of time (Sohn et al., 2009). The nodular bronchiectatic form is seen in the middle lobe of the right lung and lingula in the left lung. This form typically is present in nonsmoking postmenopausal women as bilateral bronchiectasis with small nodular opacities and tends to have a much slower course over time (Sohn et al., 2009; Moon et al., 2019). The natural course is usually indolent but progressive with a decline in pulmonary function, impaired quality of life and death in 15% of patients (Piersimoni and Scarparo, 2019). *MABC* infects patients with CF at an early age, and accounts for half of NTM isolates (Harris et al., 2012; Ravnholt et al., 2018). In CF patients, infection leads to an unexpected decline in lung function. This infection is particularly important in adolescents as it can cause the largest loss of potential years of life (Ravnholt et al., 2018).

### Resistance to Antibiotics

Resistance updates reveal that the molecular mechanism responsible for macrolide resistance is the expression of an erythromycin ribosome methylase, *erm* gen. Other resistance molecular mechanisms include the *rrs* gene responsible for resistance in aminoglycosides, a class A beta-lactamase associated with resistance to most Beta-lactams (only cefoxitin and imipenem are useful), and enzymatic inactivation of most tetracyclines (except tigecycline). The principal mechanisms of antibiotic resistance are described in **Table 2**.

**Table 2:** Mechanism responsible for MABC antimicrobial resistance.

ANTIMICROBIAL	MECHANISMS OF RESISTANCE	ENZYME/GENE	LOCATION
**Aminoglycosides**	Target modifying enzymes	Aminoglycoside2- N –acetyltransferase and aminoglycosies phosphotransferases^a^	* *
**Deoxystreptamine Aminoglycosides**	Acquired resistance by point gene mutations	*rrs* gene encoding 16S rRNA protein	Mutations include T1406A, Cl409T, A1408G, and G1491T.^b^
**Beta Lactams**	Antibiotic degrading enzymes	β-lactamase encoding genes.	Class A β-lactamase Bla_Mab (MAB_2875)
**Macrolides**	Target modifying enzymes Acquired resistance by point gene mutations	Functioning erythromycin ribosome methylase *erm(41)* gene rrl gene encoding 23S rRNA transferase	Reversion to susceptibility: 274 bp deletion at positions 159- 432 and T28C point mutation.^C^ Point mutations at positions 2058 and 2059
**Fluoroquinolones**	Polymorphism in target genes	Nucleotide variation at the Quinolone resistance determining region in the DNA gyrase- GyrA – GyrB^d^	Ala-90 gyrA gene Arg-516 and Asp-533 gyrB gene
**Tetracyclines**	Enzymatic inactivation	Flavin- adenine- dinucleotide (FAD)- inactivating monooxygenase (MabTetX)	

a Twelve putative aminoglycoside phosphotransferases are encoded within the MABC genome, which could contribute to resistance to this group of antibiotics (Nessar et al., 2012; Luthra et al., 2018).

b Mutations associated with aminoglycoside resistance (Ananta et al., 2018).

c These mechanisms are associated with reversion to clarithromycin susceptibility (Nie et al., 2014; Zhu et al., 2015). A T28C point mutation (thymidine to cytosine polymorphism at the position 28) results in tryptophan to arginine amino acid change at codon 10, rendering a non-functional erm 41 gene (Pavan et al., 2017), C28 polymorphism is related to susceptibility (Luthra et al., 2018).

d Quinolone resistance is associated with gyrA and gyrB mutations, a previous study showed all resistant isolates encoded the same amino acids in the quinolone resistance determining region (Kim et al., 2016).

The mechanism responsible for inducible macrolide resistance is the expression of an erythromycin ribosome methylase, *erm(41)* gene, in the presence of macrolides (Koh et al., 2014). This methylase transfers one or two methyl groups to adenine in the peptidyl region of 23S rRNA, preventing clarithromycin binding (Nessar et al., 2012; Pavan et al., 2017). *M. massiliense* harbors a truncated *erm(41)* gene being intrinsically susceptible to clarithromycin whereas *M. abscessus* contains a complete *erm(41)* conferring resistance, *M. bolletti* has the T28 polymorphism and may develop resistance to macrolides during therapy (Nessar et al., 2012; Kim et al., 2016; Pavan et al., 2017). Mutations in the *rrl* gene encoding the 23S rRNA peptidyl transferase are also associated with acquired macrolide resistance (Choi et al., 2017).

Spontaneous single mutations in the *rrs* gene encoding the 16S rRNA are responsible for resistance to aminoglycosides (Nessar et al., 2012). Nucleotide variation at the quinolone resistance determining region (QRDR) confers resistance to fluoroquinolones (Kim et al., 2016; Johansen et al., 2020). The genome of *MABC* encodes a class A β-lactamase (Bla_Mab_) which is associated with resistance to most β-lactams. *MABC* hydrolyzes several members of cephalosporins and carbapenems, reducing its activity (Luthra et al., 2018; Story-roller et al., 2019). Cefoxitin and imipenem have moderate activity *in vitro* against *MABC* as they are hydrolyzed at a slow rate *via* Bla_Mab_ (Johansen et al., 2020).

Enzymatic inactivation of tetracyclines by the Flavin- adenine- dinucleotide (FAD)- inactivating monooxygenase (MabTetX) has been reported (Rudra et al., 2018). This change is associated with high levels of resistance to doxycycline; however, tigecycline resists inactivation explaining its good activity *in vitro (* Luthra et al., 2018).

### Diagnosis

Due to difficulties in differentiating between colonization from *MABC* isolates and true disease, clinical, radiologic, and microbiologic criteria are required for diagnosis (Ryu and Daley, 2016). The American Thoracic Society (ATS) and Infectious Diseases Society of America (IDSA) clinical guidelines for NTM diagnosis include these considerations.

A direct smear of the respiratory specimen should be examined using a fluorescent method with higher sensitivity than conventional stains like Ziehl-Neelsen; however, microscopy alone cannot distinguish between mycobacterial species and viable or non-viable specimens (Somoskovi, 2014). Several types of microbiologic culture systems have been used for diagnosis of RGM and *MABC*, including growth indicator tube MGIT with BACTEC MGIT 960. Diagnostic updates suggest that culture is mandatory in all cases for diagnosis (Cortes et al., 2010). Growth is necessary for precise identification, with molecular methods being the gold standard for identification (Jones et al., 2019).

Despite identical 16S RNA genes, accurate identification can be achieved with the use of different targets that include RNA Polymerase *(rpoB), gyrB*, heat shock protein (*hsp65)*, internal transcribed spacer *(ITS)*, superoxide dismutase *(sodA)* and 16S - 23S rRNA gene spacer amplification (Somoskovi, 2014; Jones et al., 2019). Mass spectrometry (MALDI- TOF) is capable of distinguishing between *M*. *chelonae* and *MABC* but cannot differentiate between the three subspecies of *MABC* (Jones et al., 2019).

Genotyping methods can be used to assess strain relatedness allowing outbreaks recognition. Commonly used methods are pulsed-field gel electrophoresis (PFGE), multilocus sequencing, and methods based on minisatellite sequences (variable number tandem repeats VNTR) and amplified fragment length polymorphism (Somoskovi, 2014). PFGE has been the standard method for differentiating strains within the complex (Howard, 2013). WGS offers a higher degree of resolution than other genotyping methods providing crucial information about transmission events (Harris and Kenna, 2014).

### Treatment

Therapeutic updates suggest that *in vitro* antimicrobial testing in broth microdilution assay is required; incubation for 14 days is necessary to rule out inducible resistance for macrolides (Somoskovi, 2014; Jones et al., 2019).

Clinical and Laboratory Standards Institute (CLSI) guidelines recommend minimal inhibitory concentration (MICs) for susceptibility testing using a panel of 10 antimicrobials: amikacin, cefoxitin, clarithromycin, ciprofloxacin, doxycycline, imipenem, linezolid, moxifloxacin, trimethoprim-sulfamethoxazole, and tobramycin (Koh et al., 2014).

*MABC* is one of the most resistant NTM, being uniformly resistant to conventional antituberculous drugs (Nessar et al., 2012; Howard, 2013). Cure rate is low, with success rates between 30 to 50% (Nie et al., 2014), except for *M. massiliense* (80 to 90% success rate) (Choi et al., 2017). A study on the clinical impact of differentiating *M. massiliense* and *M. abscessus* showed that clinical and radiological findings caused by the subspecies are very similar; however, the sputum conversion and maintenance of negative sputum was higher in patients with *M. massiliense* (88%) pulmonary disease than *M. abscessus* (25%) disease with overall negative cultures in 58% of patients (Koh et al., 2011). Different sputum conversion rates could be explained by local differences in the prevalence of subspecies (Pasipanodya et al., 2017).

Treatment of *MABC* requires 18 months of multidrug therapy (Story-roller et al., 2019). Multidrug regimen includes macrolide based combination therapy with two parenteral agents for the initial phase (Jeong et al., 2017; Pasipanodya et al., 2017). Initial therapy should be given for at least 2-4 months, followed by oral macrolide based therapy (Koh et al., 2014; Lee et al., 2015). Recently, the British thoracic society has recommended for the initial phase to include 4 weeks of intravenous amikacin, tigecycline, imipenem (when tolerated) and oral clarithromycin. For the continuation phase, nebulized amikacin and an oral macrolide with one to three of the following: linezolid, clofazimine, minocycline cotrimoxazole, and moxifloxacin (Haworth et al., 2017). The goal is twelve-month sputum culture negativity (Thomson and Yew, 2009); However, recurrence and drug-related toxicity secondary to long term therapy are frequent, making this outcome unrealistic for many patients (Lyu et al., 2011; Koh et al., 2014). One study evaluating outcomes in patients with long term injectable therapy showed adverse effects in 43.9% of the patients, the most common was drug-induced liver injury (Lyu et al., 2011). Alternative goals include symptomatic improvement and radiographic regression of infiltrates rather than sputum conversion (Griffith et al., 2007; Koh et al., 2014).

A combination of oral clofazimine and inhaled amikacin demonstrated to be effective in refractory *MABC-PD (* Choi et al., 2017). Tigecycline combined with clarithromycin also appears synergistic against *MABC* (92.9% of patients with *MABC* infection). Additionally, tigecycline has shown good tissue penetration and few severe side effects making long-term therapy with this antibiotic an option (Huang et al., 2013). Other antibiotics such as linezolid showed good activity in clinical trials (Zhang et al., 2018). Rifabutin exhibits *in vitro* activity against *MABC (* Luthra et al., 2018). Bedaquiline is also active against *MABC* isolates (Johansen et al., 2020). A recent study showed synergy between non-β-lactam based inhibitors (avibactam, vaborbactam) with β-lactams, avibactam lowered the MICs for several β-lactams by 4-32 fold (Luthra et al., 2018).

Recently, a study showed that clarithromycin-nanocapsules reduced colony numbers during *MABC* infection as they improved delivery of antimicrobial to mycobacteria inside macrophages (Anversa Dimer et al., 2020). NO has been also used as a promising strategy for treatment in patients with *MABC* infection and CF as NO kills bacterial cells and disperse biofilms avoiding resistance (Chiarelli et al., 2020). A young patient with CF and disseminated *MABC* infection was treated successfully with a three- phage cocktail therapy after lung transplantation (Dedrick et al., 2019). These new therapies represent promising strategies to overcome *MABC* resistance.

## Conclusion

*MABC*-PD has become a significant cause of pulmonary infection. When isolated, it is likely to represent a real infection. Precise identification to subspecies level is mandatory considering the differences in antibiotic susceptibility profiles and treatment outcomes; differentiation has become possible due to the development of new microbiological and molecular techniques. Macrolides are the mainstay of therapy; therefore, identification of inducible resistance strains is necessary before starting treatment. *MABC* is considered a multidrug-resistant pathogen, and increasing resistance is highly likely to continue given the limited existing therapeutic options. Consequently, it is important to develop novel therapies and identify combinations of currently available antimicrobials to improve treatment outcomes.

## Author Contributions

LV, AG, JG, and JR conceived of the present idea and performed literature review. LV wrote the manuscript with the support from JG and JR. All authors contributed to the article and approved the submitted version.

## Conflict of Interest

The authors declare that the research was conducted in the absence of any commercial or financial relationships that could be construed as a potential conflict of interest.
